# Assessment of episodes of pneumonia and diarrhea in vaccinated and unvaccinated children under 60 months of age

**DOI:** 10.12669/pjms.36.7.2996

**Published:** 2020

**Authors:** Shireen Qassim Bham, Farhan Saeed, Muhammad Athar Khan, Rashid Naseem Khan

**Affiliations:** 1 Dr. Shireen Qassim Bham, FCPS. Department of Pediatrics, Liaquat College of Medicine & Dentistry, Karachi, Pakistan; 2 Dr. Farhan Saeed, FCPS. Department of Pediatrics, Liaquat College of Medicine & Dentistry, Karachi, Pakistan; 3 Dr. Muhammad Athar Khan, MCPS, DPH, MBA, PGD Statistics, DCPS-HCSM, DCPS-HPE, CCRP Department of Community Medicine, Liaquat College of Medicine & Dentistry, Karachi, Pakistan; 4 Dr. Rashid Naseem Khan, MCPS, MD. Department of Medicine, Liaquat College of Medicine & Dentistry, Karachi, Pakistan

**Keywords:** Diarrhea, Pneumonia, Rota virus, Pneumococcal vaccine

## Abstract

**Objective:**

To assess the episodes of pneumonia and diarrhea in vaccinated and unvaccinated children under 60 months of age.

**Methods:**

This descriptive cross-sectional study was carried out at Darul Sehat Hospital and SESSI, Karachi in the Department of Pediatrics from 1^st^ November 2018 to 3^rd^ February 2019. An interview based questionnaire was administered and selection of participants was done by convenience sampling. Total of 196 participants were selected for interview.

**Results:**

Total 196 participants were interviewed which included mothers of children between the age group of 1- 60 months. The questions were entered on the questionnaire after taking consent from the mothers. Among them, males were 98 (52.7%) and females were 88(47.3%). One hundred seventy two (88.7%) children were vaccinated for pneumococcal and rotavirus whereas unvaccinated children were 22(11.3%). There was an incidence of 66(63.5%) for loose watery diarrhea. In vaccinated children, grading of diarrhea was found to be severe cases as 30 (34.9%), moderate cases as 27 (31.4%) and mild cases as 29(33.7%). For unvaccinated children, severe cases were 12 (66.7%) and for mild and moderate cases were (11.1%) and 04(22.2%) respectively with p value of 0.035. As compared to their unvaccinated counterparts, the frequency of severe pneumonia was far less than cough and cold in vaccinated children (p<0.001).

**Conclusion:**

There is significant reduction in cases of severe pneumonia in children receiving pneumococcal vaccine as compared to children receiving Rota vaccine there is moderate reduction in cases of severe diarrhea. The overall coverage of Pneumococcal and Rota vaccines was higher in our sample population. Efforts should be made to increase the awareness of Rotavirus vaccination in order to have better coverage in future.

## INTRODUCTION

In children less than 60 months of age, pneumonia and diarrhea are one of major reasons of morbidity and mortality and main reason for high economic cost.^1^ Pneumonia is the major cause of childhood mortality globally with 0.7 to 1 million death of children under the age of 60 month annually.^2^ Pneumonia lead to 935,000 deaths in children globally in 2013.^3^ Streptococcus pneumonia is the pathogen mostly affecting infants, causing an estimated killing of 1.2 million worldwide.^2^ In developing countries pneumonia accounts for 15% of total overall mortality in children under five years of age worldwide.^4^ It has been estimated that 29% of all diarrheal deaths in children <5 years of age is due to rotavirus and about 23% of rotavirus deaths are in the Indian subcontinent.^4^ Studies have estimated that specific PCV formulations could reduce overall under-five mortality by 11%.^2^

In young children, Rotavirus is a leading cause of diarrhea. Rotavirus infection was responsible for more than 258 million episodes of diarrhea among children younger than 60 months.^5^ Rotavirus accounts for 29% of overall deaths due to diarrhea in children under-60 months of age with 23% of deaths due to rota virus occurring in developing countries like Indo Pak subcontinent.^6^ Annually Rotavirus is responsible for >500,000 deaths worldwide among infants and very young children, with 90% of these deaths occurring in African and Asian countries alone. Rotavirus infection alone accounts for 40% of all pediatric hospitalizations for diarrhea. Hence other than clean water usage, personal hygiene and good medical care, rotavirus vaccine is considered to be a safest strategy to decrease the burden of severe and fatal rotavirus diarrhea.^7^

There are limited studies on diarrhea and pneumonia after the introduction of pneumococcal and Rota virus vaccine so further work is needed in this context. Also limited number of studies from a developing countries to report the frequency of both diarrhea and pneumonia in vaccinated and unvaccinated children in a well-defined catchment area. In this study we assessed vaccinated and unvaccinated children for the frequency of pneumonia and diarrhea in children under the age of 60 months at tertiary care hospital, Karachi.

## METHODS

This descriptive cross-sectional study was, carried out at Darul Sehat Hospital and SESSI after the ethical approval (Ref. No. DSH/IRB/2020/0014, dated May 5, 2020), Karachi in the Department of Pediatrics from 1^st^ November 2018 to 3^rd^ February. The minimum sample size came out 196 by using Raosoft sample size calculator. An interview based questionnaire was administered and selection of participants was done by convenience sampling. All children age one month to 60 months coming to outpatient clinic with their mothers for any illness were included in this study. A written informed consent was taken from parents. The participants were asked for age, gender, number of vaccines received, type of vaccines, duration of illness, history of previous episodes, breastfeeding, reason for vaccination, reason for no vaccination which were taken as variables.

Diarrhea is defined as stools >3 episode in 24 hours. Mild Diarrhea when child had no visit to health care subsided itself or with home remedies, moderate when child visited primary health care /clinic for treatment and rehydration performed orally at home and severe when child visited emergency department for treatment at hospital.^8^,^9^

WHO IMNCI guidelines were used for diagnosis on pneumonia are as follows: Cough and cold when child has runny nose and cough but no fast breathing. Pneumonia when child had fast breathing and taken to hospital and Severe pneumonia when child has chest in drawing or any danger signs (reluctant to feed/unconscious, vomiting every feed, having convulsions).^10^

Data was entered and analyzed using SPSS version 21. The frequency (%) and mean+SD were reported for qualitative and quantitative variables. Chi Square test was used to compare severity of diarrhea and pneumonia in vaccinated and unvaccinated children at p value <0.05 significant.

## RESULTS

One hundred ninety-six participants were interviewed which included mothers of children between the age group of 1- 60 months. The questions were entered on the questionnaire after taking consent from the mothers. Among them, males were 98 (52.7%) and females were 88(47.3%). One hundred seventy-two (88.7%) children were vaccinated for pneumococcal and rotavirus whereas unvaccinated children were 22(11.3%). There was a frequency incidence of 66(63.5%) for loose watery diarrhea.

**Fig.1:**
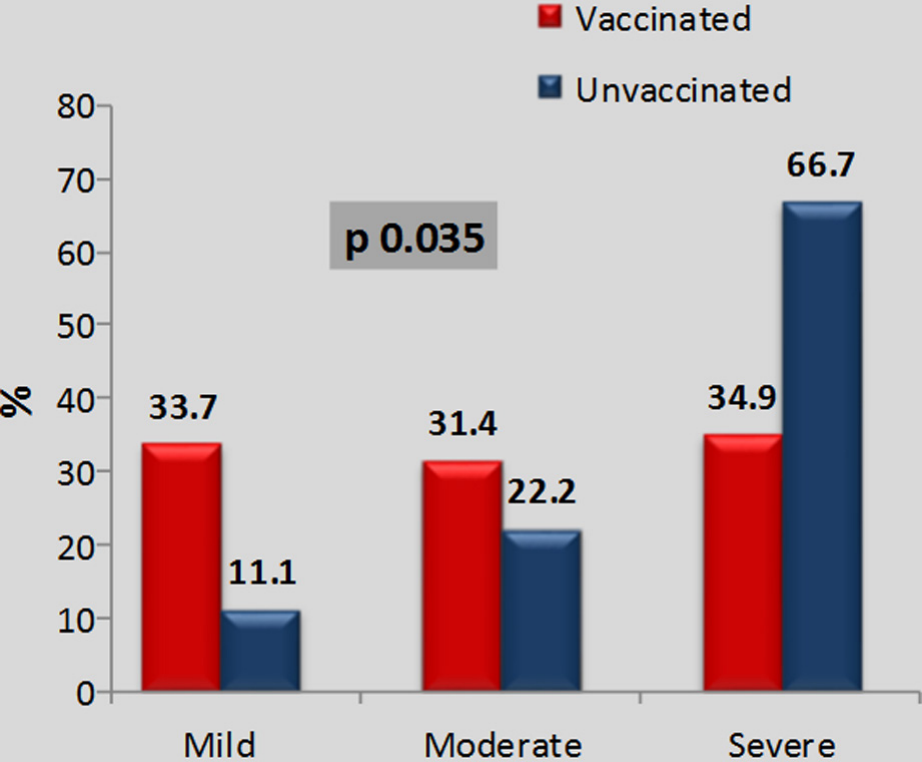
Severity of Diarrhea in Vaccinated and Unvaccinated Children According to IMNCI.

The Fig.1 shows the frequency of diarrhea according to its severity in vaccinated; mild 29(33.7%), moderate 27 (31.4%), severe 30 (34.9%), and unvaccinated participants; mild 02 (11.1%), moderate 04 (22.2%), severe 12 (66.7%) accordingly.

As compared to their unvaccinated counterparts, the frequency of severe pneumonia (16.9%) was far less than cough and cold (63.1%) in vaccinated children (p<0.001). Fig-II

**Fig.2:**
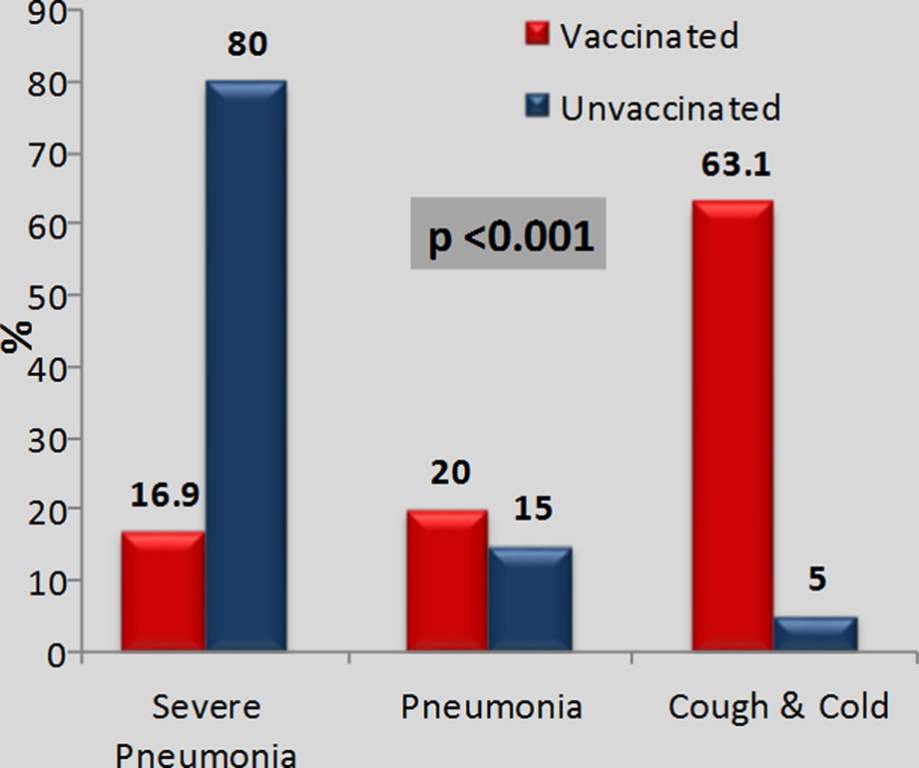
Severity of Pneumonia in Vaccinated and Unvaccinated Children According to IMNCI.

## DISCUSSION

In Children age less than 60 months of age worldwide; gastrointestinal and respiratory infections are the two leading causes accounting for over two million deaths annually. In our study overall vaccination coverage was 88.7% for both Rota and Pneumococcal vaccines. In India, Rota vaccine coverage was 65% in 2016 and 61% in 2017^11^ where as in Canadian study the coverage was 75 to 90% in different cities.^12^ In another Indian study, pneumococcal coverage was 64% for PCV10 and 74.6% for PCV 13.^13^ In other studies, Brazil shows overall vaccination coverage of 53.4%^14^ and in Uganda it was 42%^15^ both had far less coverage than in our study. Overall Global vaccination coverage according to WHO remains at 85%, global coverage for pneumococcal was estimated at 44% and for Rota it was estimated at 28%.^16^ Comparison to these studies shows that the coverage in our part of world is comparatively better to neighboring countries.

**Table-I:** Demographic and clinical characteristics of study participants (n=196).

Variables		n (%)
Gender	Male Female	98(52.7) 88(47.3)
Vaccine received Rota, pneumococcal	Both None	172(88.7) 22(11.3)
Age in months	Mean ± SD, Median (min, max)	25.6±18.3, 24(1-60)
Grading of pneumonia	Severe pneumonia	38(25.3)
	Pneumonia	29(19.3)
	Cold and cough	83(55.4)
History of previous episodes of pneumonia	Yes	130(68)
	No	61(32)
Grading of diarrhea	Mild	31(29.8)
	Moderate	31(29.8)
	Severe	42(40.4)
History of previous episodes	Yes	88(48)
	No	95(52)
Reason of vaccination	Health concern	50(36.7)
	Knowledge	86(63.3)
Reason of no vaccination	Fear of side effects	19(32.3)
	No knowledge	16(27.1)
	Cost	6(10.1)
	Other	18(30.5)
Type of diarrhea	Acute Watery diarrhea	66(63.5)
	Diarrhea with mucous	20(19.2)
	Blood in stool	2(1.9)
	Other	16(15.4)
Breastfeeding	Yes	169(88.4)
	No	22(11.6)

**Table-II:** Duration of Illness of Pneumonia and Diarrhea.

Variables	Mean± SD, Median(Min-Max)
Duration of Illness of Pneumonia	7.1±6.4,5(1-30)
If Yes, At What Age? (Pneumonia)	9.7±13.2,4(1-60)
Duration of Illness of diarrhea	6±8.1,4(0-48)
If Yes, At What Age? (Diarrhea)	10.2±12.6,6(1-60)

Our study shows mean age of 10 months with median age of 06 month (1-60) which is similar to paper reviewed by WHO which shows median age in less developed countries as 06 -09 months.^7^ Whereas in India the peak age is between 9 to 11 month^16^ and 6 to 15 months in another Indian study.^17^ There were 52.7% were male and 47.3% were female with 1:1 male to female ratio in our study while study done in India shows M:F ratio of 0.97:1^18^ with female predominance.

The frequency of watery diarrhea in our study was 63.4%. the incidence of diarrheal disease in study done in Nigeria children under five years was 51.8%.^19^ A peak incidence of 1.05(95% confidence interval [CI]: 0.64, 1.64) infections per child-year was observed in the first six months of life in Vellore, while in Karonga incidence was greatest in the second six months of life (1.41 infections per child year [95% CI: 0.79, 2.29]).^20^ In unvaccinated children, severe diarrhea was the most prevailing one with 66.7% with p value being 0.035. In vaccinated children, mild diarrhea was seen in 33.7% moderate diarrhea in 31.4% and severe diarrhea in 34.9% children in comparison to study done in India which had 73.7% as moderately severe disease and 26.3% as severe disease. There was no case of mild disease.^17^ In other study with children under-five, it showed 64.8% of diarrheal episodes are mild, 34.7% are moderate, and 0.5% are severe.^21^ This clearly shows Rota virus vaccination significantly decreases the burden of disease in community and is effective in preventing severe rotavirus gastroenteritis.

Breast feeding has an important role in prevention of Rota virus gastroenterits thus reducing the risk of subsequent Rota virus infection. Our study shows, 88.4% children were given breast feeding while in Indian study it was 46.4%.^18^ In study by Sushmita Das, the prevalence of breastfed infants to non breast feed infants with diarrhea was 23.4% as compared to 76.5%.^22^ This shows breast feeding has a protective effect against diarrhea. Effective measure like use of clean water and effective personal hygiene along with breast feeding and proper doses of Rota virus vaccination leads to corresponding decline in Rota virus gastroenteritis.

In our study mean age for children with pneumonia is 7.1 months as compared to study done in Dhaka which shows 11 months^23^ and median age 2.4 months as compared to a multicenter study by Thomas Bennet, Valentine Sanchez et al which shows median age is 14 months.^24^ In our study frequency of severe pneumonia is 25.3%, pneumonia 29% and cold and cough 55.4% as compared to study in Kenya which shows 74% were cough and cold and 22% were severe pneumonia.^25^ In vaccinated children severe pneumonia decrease to 16.9% and cough and cold increased to 63.1% in our study which is proved by study in Gambia that states vaccine effectiveness increase with larger number of doses.^26^

The reports clearly show that after the introduction of PCV10/13 vaccine there is significant evidence of reduction in hospitalization for pneumonia with an estimated decline in the incidence of radiological pneumonia to 24% and 47% in studies done in Gambia^26^ and Israel^27^ respectively. The addition of Pneumococcal vaccine to EPI is an essential milestone in the fight against pneumonia. The use of this vaccine will not only reduce significant number of new cases of pneumonia but has great potential to save thousands of lives.

### Strengths of the study

The strength of our study is despite unavailability of data to study combine effect of Rota virus and pneumococcal vaccine, we tried to evaluate the effect of these vaccines in children less than 60 months of age in Pakistan. It has proven to be cost effective by reducing the disease burden.

### Limitations of the study

Because of short duration we could not asses the long-term effect.

## CONCLUSION

There is significant reduction in cases of severe pneumonia in children receiving pneumococcal vaccine as compared to children receiving Rota vaccine while there is moderate reduction in cases of severe diarrhea. The overall coverage of Pneumococcal and Rota vaccines was higher in our sample population. Efforts should be made to increase the awareness of Rotavirus vaccination in order to have better coverage in future.

### Authors’ Contribution:

**SQB, FS:** Study design, data collection, Manuscript preparation, Review and final approval of manuscript and is responsible for integrity of research.

**MAK:** Statistical analysis& interpretation, Drafting, Manuscript preparation.

**RNK:** Data collection, Manuscript preparation, Review and final approval of manuscript.
